# Randomized controlled trial of effects of metformin in NAFLD patients with newly diagnosed type 2 diabetes treated with an intensive lifestyle: a study protocol

**DOI:** 10.1186/s13063-025-09191-0

**Published:** 2025-10-31

**Authors:** Ke Chen, Chuyin Zhong, Lixin Zhou, Liting Cao, Danhua Liang, Lan Liu, Yongqian Liang

**Affiliations:** The Eighth Affiliated Hospital of Southern Medical University, (The First People’s Hospital of Shunde), Foshan City, Guangdong Province China

**Keywords:** Metformin, Calorie-restricted dietary, Exercise, Glycemic control, NAFLD, T2DM

## Abstract

**Background:**

Nonalcoholic fatty liver disease (NAFLD) and type 2 diabetes (T2DM) are common chronic diseases. The coexistence of NAFLD and diabetes accelerates the progression of both and tends to induce bad prognosis. To date, lifestyle modification has been suggested as the first-line treatment for NAFLD patients. Some studies indicated that lifestyle modification could normalize glucose levels in newly diagnosed T2DM, but many individuals do not reach glycemic targets in practice using lifestyle modification alone. Considering the tight link between NAFLD and T2DM, metformin, a widely used drug in diabetic patients with a good safety profile, may be a possible treatment or control of NAFLD progression. There is no therapeutic consensus for the treatment of NAFLD patients with diabetes. Therefore, it is essential to elucidate the beneficial effects of lifestyle modification combined with metformin on glucose-lipid metabolism in the NAFLD population with newly diagnosed T2DM.

**Methods:**

Participants will be recruited in this single-center, randomized clinical trial through telephone interview. All participants will be assigned randomly at a 1:1:1 ratio to three groups and treated with lifestyle modification including calorie-restricted dietary and exercise therapy, 500–2000 mg/day metformin, or treated with both lifestyle modification and 500–2000 mg/day metformin (the co-intervention group) for 12 weeks. The primary outcomes are changes in glycemic control and severity of fatty liver from baseline to 12 weeks of follow-up.

**Discussion:**

This trial is designed to clarify whether lifestyle intervention combined with metformin has synergistic effects on glycemic control, attenuating the progression of NAFLD in NAFLD patients with newly diagnosed T2DM.

**Trial registration:**

ChiCTR2300072031. Registered on 31 May 2023.

**Supplementary Information:**

The online version contains supplementary material available at 10.1186/s13063-025-09191-0.

## Administrative information

Note: The numbers in curly brackets in this protocol refer to SPIRIT checklist item numbers. The order of the items has been modified to group similar items (see http://www.equator-network.org/reporting-guidelines/spirit-2013-statement-defining-standard-protocol-items-for-clinical-trials/).

**Table:** 

Title {1}	Randomized controlled trial of effects of metformin in NAFLD patients with newly diagnosed type 2 diabetes treated with intensive lifestyle: a study protocol
Trial registration {2a and 2b}	ChiCTR2300072031
Protocol version {3}	Version 2, 16 May 2023
Funding {4}	This study is supported and funded by Scientific Research Start Plan of Shunde Hospital, Southern Medical University (Grant code: CRSP2022010) and the Medical Science and Technology Research Project of Foshan (grant code: 2320001006951)
Author details {5a}	Ke Chen and Yongqian Liang: methodology and approved the final manuscript; Ke Chen: wrote and submitted the manuscript. Chuyin Zhong, Lixin Zhou, Liting Cao, Danhua Liang, and Lan Liu: participant recruitment and data curation. All authors have read this manuscript and consent the submitted version
Name and contact information for the trial sponsor {5b}	Yongqian Liang, MM, Shunde Hospital, Southern Medical University (The First People's Hospital of Shunde), No. 1 Jiazi Road, Lunjiao, Shunde District, Foshan City, Guangdong Province, China E-mail: 102lyq@smu.edu.cn
Role of sponsor {5c}	The funders do not play a role in the design of the study and collection, analysis, and interpretation of data and in writing the manuscript

## Introduction {6a}

Nonalcoholic fatty liver disease (NAFLD) is the most common chronic disease, with increasing incidence worldwide [1]. Available evidence indicated that NAFLD is closely associated with type 2 diabetes, most likely due to a common pathophysiological mechanism—insulin resistance [2]. A recent meta-analysis showed that patients with NAFLD have twofold higher increased risks of developing T2DM [3]. Similarly, more than 55% of patients with T2DM would develop NAFLD [4]. The coexistence of NAFLD and diabetes accelerates the progression of both and worsens hepatic and extrahepatic outcomes [5]. Thus, the early detection of NAFLD in newly diagnosed T2DM patients is of great clinical significance.

To date, there is no approved medication or surgery to treat NAFLD patients. Lifestyle could be the first-line intervention in patients with NAFLD. The efficiency of lifestyle modification on NAFLD has been elucidated in research [6]. Available evidence indicated that the biochemical remission of newly diagnosed diabetes is achievable through intensive lifestyle modification [7]. But the clinical intervention effects are inconsistent.

Metformin, as the first-line pharmacological therapy of T2DM, is prior to or in parallel with lifestyle modifications for the treatment of newly diagnosed diabetes. To our knowledge, metformin has been shown to have beneficial effects in alleviating hepatic lipogenesis in animal models of NAFLD. Zhang R. et al. revealed that metformin is effective in reducing body mass index (BMI), liver fat content, liver enzymes, and hemoglobin A1c (HbA1c) and improved insulin resistance in NAFLD patients with T2DM [8]. However, there are conflicting reports. In a prospective study, metformin was not associated with improvements in transaminase levels, hepatic fat content, and HbA1c [9]. Given the widely used metformin in diabetes patients, the beneficial effects of metformin treatment need to be further elucidated among NAFLD patients with T2DM. The objective of this randomized clinical trial is to test the efficacy of metformin as an adjuvant to lifestyle modification in improving glucose-lipid metabolism among NAFLD patients with newly diagnosed T2D.

### Objectives {7}

The primary objective of this randomized clinical trial is to evaluate the efficacy of lifestyle modification combined with metformin in NAFLD patients with newly diagnosed type 2 diabetes.

### Methods/design {8}

The study is a randomized, single-center, parallel group, superiority clinical trial. All patients who meet eligibility criteria are invited to this study and then assigned to different groups with a 1:1:1 allocation rate, receiving 12 weeks of follow-up.

## Methods: participants, interventions and outcomes

### Study setting {9}{15}

**Fig. 1:**
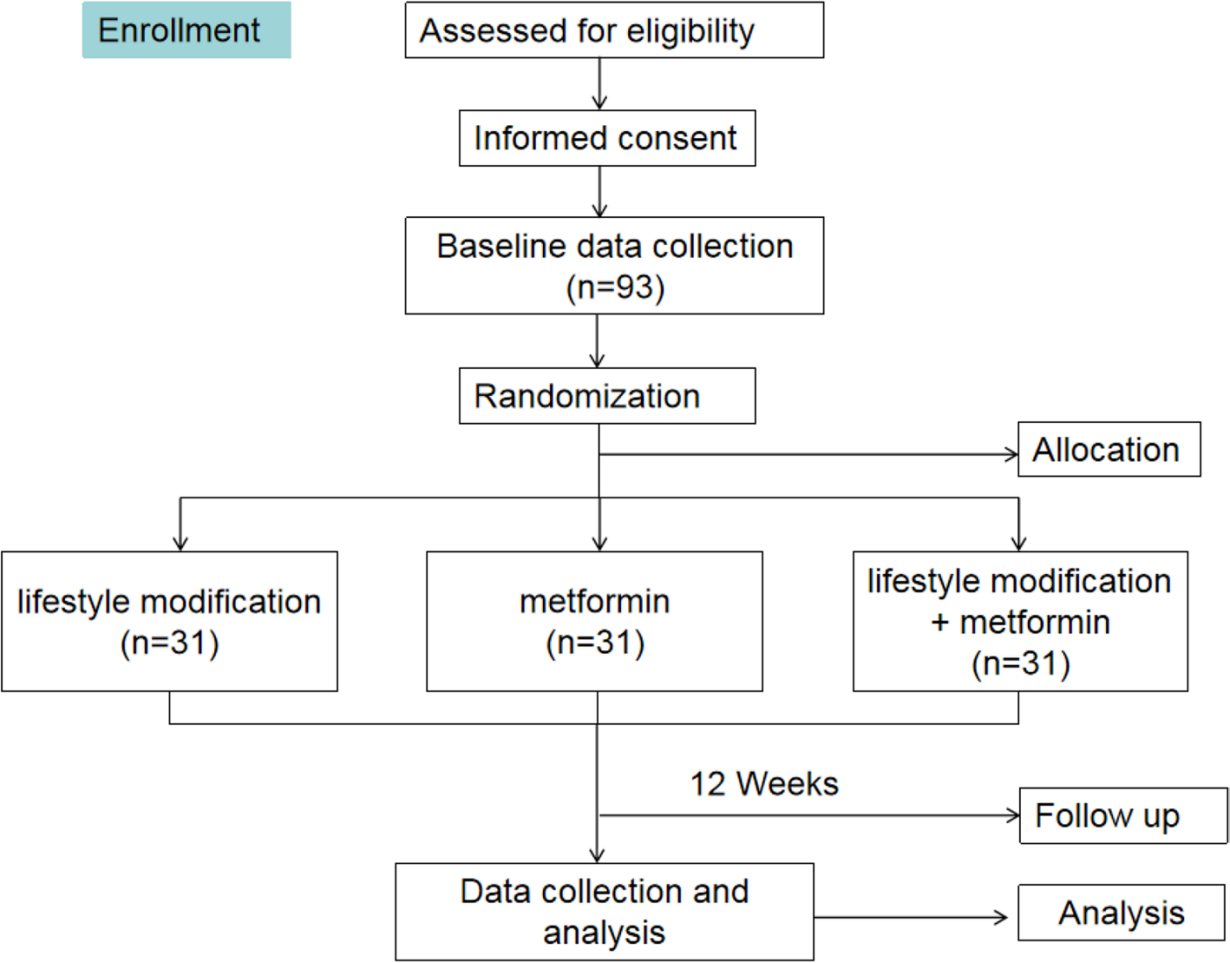
The trial flow diagram

This randomized clinical trial will be conducted in Shunde Hospital of Southern Medical University, a tertiary hospital in Foshan, China. Participants will be recruited through healthcare centers and advertising in media. All subjects are the newly diagnosed diabetes with NAFLD. The study protocol was granted by the Ethics Committee of Shunde Hospital of Southern Medical University (reference number: KYLS20220615) and was registered at the Chinese Clinical Trials Registry (ID: ChiCTR2300072031) prior to the start of recruitment. Enrolment of participants commenced in June 2023. The study design flow diagram is presented in Fig. 1.

### Participant characteristics and eligibility criteria {10}

The inclusion criteria were as follows: (1) Men and women aged 18–65 years, (2) diagnosed with NAFLD, (3) newly diagnosed T2DM (diagnosed with T2DM within 3 years) and HbA1c ≤ 10.0%, (4) body mass index (BMI) > 23.9 kg/m^²^, and (5) all participants would provide written and oral informed consent before any testing. Exclusion criteria were as follows: (1) Habitual alcoholic intake (alcohol consumption > 30 g/day and > 210 g/week in men and > 20 g/day and > 140 g/week in women); (2) liver disease: ALT/AST thrice normal range, diagnosis of viral hepatitis, autoimmune liver disease, liver cirrhosis, and drug-induced hepatotoxicity; (3) the presence of one or more micro- and macrovascular complications of T2DM; (4) diseases that affect blood glucose levels (e.g., hyperthyroidism and hypercortisolism) or acute diabetic complications (e.g., ketoacidosis and hyperosmolar coma) or type 1 diabetes; (5) taking anti-fatty liver, anti-liver fibrosis drugs, drugs affecting glucose and lipid metabolism, and related health products; and (6) pregnant and lactating women.

### Who will take informed consent? {26a}

All subjects will be provided written informed consent before participating and then randomly assigned to three groups for a 3-month intervention. The informed consent will be collected by the treatment doctor.

### Additional consent provisions for collection and use of participant data and biological specimens {26b}

A voluntarily stool sample will be collected for analysis of intestinal flora for all subjects.

## Interventions

### Explanation for the choice of comparators {6b}

A lifestyle modification plays a fundamental role in the management of patients with newly diagnosed T2DM or NAFLD. However, many diabetic patients or NAFLD patients fail to achieve clinical goals in clinical practice. Metformin is the most common anti-hyperglycemic agent. The protective effect in chronic liver disease is controversial. We want to further clarify whether there is a synergistic reaction between lifestyle modification and metformin in NAFLD patients with newly diagnosed type 2 diabetes.

### Intervention {11a}

Participants will be randomly allocated to three groups according to the table of random numbers. Participants in the group of lifestyle modification receive a calorie-restricted dietary intervention (1200–1400 kcal/day for males, 1000–1200 kcal/day for females) and an exercise intervention including at least 150 min of moderate intensity exercise training per week. Participants in the group of metformin receive metformin at a dose of 500–2000 g/day and healthy education by the endocrinologist, and participants in the co-intervention group receive a calorie-restricted dietary intervention, an exercise intervention, and metformin treatment.

### Criteria for discontinuing or modifying allocated interventions {11b}

Potential adverse events mainly including hyperglycemia, hypoglycemia, and poor medication tolerance will be evaluated at the 2-week, 1-month, and 2-month phone call. Participants who refuse to contact researchers within 1 week or have severe adverse effects will be removed from the study.

### Strategies to improve adherence to interventions {11c}

To facilitate adherence to the intervention, a WeChat group which contained endocrinologists and dietitians would be established for each subject to make daily follow-up. We provide online sports courses three times a week to strengthen exercise management.

### Relevant concomitant care permitted or prohibited during the trial {11d}

The concomitant treatment with drugs or nutrients that may affect glucose-lipid metabolism is prohibited during the trial. Subjects providing stool samples are advised not to take antibiotics or probiotics. In addition to the above, there are no restrictions.

### Provisions for posttrial care {30}

All participants will no longer receive the intervention measures of this study at the end of 12 weeks. Endocrinologists will be responsible for the follow-up of all research patients, and whether to continue with blood sugar lowering treatment depends on the patient’s condition.

### Outcomes {12}

The primary outcomes are severity of NAFLD. The secondary outcomes include HbA1c, and homoeostasis model-insulin resistance index (HOMA-IR), an indicator of insulin resistance, will be calculated via formula as follows: [fasting glucose (nmol/L) × fasting insulin (mU/mL)/22.5], fasting blood glucose, 2-h postprandial blood glucose, BMI, waist circumference(WC), total cholesterol, low-density lipoprotein cholesterol, high-density lipoprotein cholesterol, triglycerides, and systolic and diastolic blood pressure.The primary and secondary outcomes will be reevaluated at the end of 12 weeks. Supplementary Table S1 presents the outcome definition.

### Participant timeline {13}

The participant timeline is shown in Table 1.

**Table 1:**
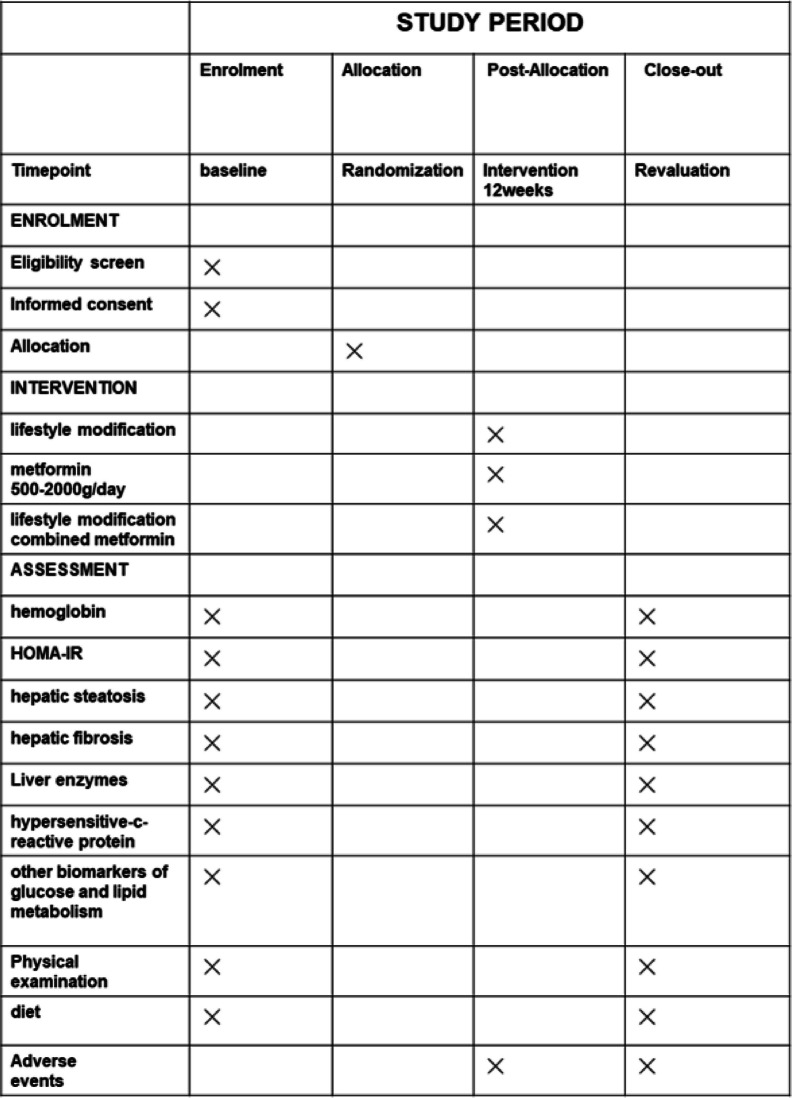
Timeline

### Sample size {14}

The sample size was calculated according to changes in the primary outcome measure (12-week change in HbA1c with *α* = 0.05, *β* = 80%). A sample size of 31 individuals per group will be selected with mean changes of 0.4 and standard deviation of 1.5 and a 20% attrition rate [10]. It is calculated by PASS 15.

## Assignment of interventions: allocation

### Random allocation sequence, concealment mechanism and implementation {16a} {16b} {16c}

Random numbers corresponded to the number of the 93 participants one by one that will be generated according to the table of random numbers. An independent researcher would arrange these random numbers from small to large. One to 31 are the lifestyle modification group, while 32–62 are designated as the metformin group, and 63–93 are the co-intervention group. The researcher responsible for recruitment will arrange subjects into the corresponding group according to the random numbers in an opaque envelope. Then, the endocrinologist and dietitian will provide the corresponding interventions.

## Assignment of interventions: blinding

### Assignment of interventions: blinding {17a} {17b}

This trial is not blinded, so unblinding will not occur.

### Plans for assessment and collection of outcomes {18a}

#### Biochemical measurements for outcomes

All laboratory analyses will be performed in a central laboratory. After a 12-h overnight fast, blood samples are collected at baseline and at 12 weeks to evaluate the biomarkers of glucose and lipid metabolism, including blood glucose concentrations measured 0 to 120 min after meal, HbA1c, fasting C peptide, fasting insulin, liver enzymes, inflammatory factor, and blood lipid.

#### Physical examination

Body weight, height, waist circumference, and hip circumference will be measured at baseline and 12 weeks later. The body composition is analyzed by InBody270, available at the hospital’s endocrinology department. BMI is calculated according to the formula. Systolic and diastolic blood pressure will be tested by a sphygmomanometer at every visit.

#### Hepatic steatosis

Transient elastography (FibroScan) would be performed after 8-h overnight fasting to assess hepatic steatosis and fibrosis according to liver stiffness measurements (LSM) and controlled attenuation parameter (CAP) score. The severity of fatty liver will be evaluated at baseline and 12 weeks later.

#### Dietary assessments

Three days of 24-h dietary questionnaire would be completed at the beginning and at the end of the study; data were collected by a professional nutritionist.

### Plans to promote participant retention and complete follow-up {18b}

To promote participant retention and complete follow-up, we will educate patients on the importance of follow-up visits of the therapy. And we make it available to all subjects to contact the principal researcher at any time and are responsible for all inspection fees and expenses for medicine.

### Data management and analysis {19}

Patient data will be recorded in case report form and then copied into Excel spreadsheets. To ensure the quality of data, we will provide training regarding data management and study-related procedures prior to the commencement of the trial to all researchers of this study.

### Confidentiality {27}

The trial and participant data will be kept confidential and stored in a secure place in Shunde Hospital, Southern Medical University (The First People’s Hospital of Shunde). All data will be entered into a computer software, using standard coding to ensure the confidentiality of the participants.

### Plans for collection, laboratory evaluation and storage of biological specimens for genetic or molecular analysis in this trial/future use {33}

Blood and stool samples are stored by the sponsor in a biobank for further research to enhance the prevention and management of diseases.

## Statistical methods

### Statistical methods for primary and secondary outcomes {20a}

All collected variables will be statistically analyzed by SPSS 28.0 statistical software and GraphPad Prism 8. Continuous variables will be assessed by analysis of covariance (ANCOVA). Categorical data will be assessed by the *χ*^2^ test. Differences between values within each arm will be evaluated using paired Student’s *t*‐tests. Nonparametric tests will be used to evaluate differences in non-normally distributed variables. Correlation analysis and multiple linear regression analysis will be used to analyze the synergistic effects of lifestyle modification and metformin. *P* < 0.05 will be used as the significance level.

### Interim analyses {21b}

No interim analyses will be performed.

### Methods for additional analyses (e.g., subgroup analyses) {20b}

There are no subgroup analyses planned.

### Methods in analysis to handle protocol nonadherence and any statistical methods to handle missing data {20c}

Data analysis will be performed according to the intention-to-treat principles. Per-protocol analyses will be done only for patients completing 12 weeks of therapy. Multiple imputation will be employed under the assumption of “missing at random.”

### Plans to give access to the full protocol, participant level-data and statistical code {31c}

The study datasets will be made available to the principal researchers on demand.

## Oversight and monitoring

### Composition of the coordinating center and trial steering committee {5d}

The trial steering committee of this project includes Ke Chen, Chuyin Zhong, Lixin Zhou, Lan Liu, Yongqian Liang, and other key research team members. All members meet periodically and collaborate in preparing all the study steps.

### Composition of the data monitoring committee and its role and reporting structure {21a}

The experimental data would be recorded by a responsible researcher. Liting Cao and Danhua Liang will review and observe each item of data.

### Adverse event reporting and harms {22}

Any discomfort during the trial should be communicated to the researcher to further assess the relevance with the trial intervention. Each adverse event will be categorized into three grades: mild, moderate, or severe. Any adverse events will be reported and documented.

### Frequency and plans for auditing trial conduct {23}

The principal researchers meet every week to assess the progress and management of the ongoing trial.

### Plans for communicating important protocol amendments to relevant parties (e.g., trial participants and ethical committees) {25}

Any modifications to the protocol, including study objectives, study design, eligibility criteria, sample sizes, or significant changes in the study, will be communicated with the ethical committee.

### Dissemination plans {31a}

The results of this work will be published via a peer-reviewed journal.

## Discussion

NAFLD is a common liver disease which includes steatosis, nonalcoholic steatohepatitis (NASH), and cirrhosis and may progress to hepatocellular carcinoma (HCC) [11]. It increases risks for T2DM [3]. Similarly, T2DM is associated with increased risks of NAFLD and contributes to the development of NAFLD [12]. Available evidence indicated that the diagnosis of T2DM was positively associated with overall mortality and liver-related outcomes in patients with NAFLD [13]. Considering the complex interrelationship, it has been suggested that NAFLD should be taken into consideration in the treatment of T2DM.

Lifestyle modification including diet and physical activity (PA) interventions is considered as first-line treatment for the treatment of NAFLD and makes it possible for remission of the disease [14]. Meanwhile, lifestyle modification plays a fundamental role in the management of patients with newly diagnosed T2DM [15]. Several dietary patterns have been studied and shown efficiency in improving glucose control [16]. Yukiko Minamiyama et al. found that calorie-restricted diets may reduce cardiovascular risk in type II diabetic rats [17]. The study by Piero Ruggenenti et al. indicated that calorie-restricted diets achieved improvements in insulin sensitivity and glomerular hyperfiltration in patients with type 2 diabetes [18]. The importance of physical activity for health has also been repeatedly underlined, and evidence has confirmed that all exercise, whether aerobic or resistance training, facilitates improved glucose regulation [19]. Studies indicated that exercise associated with calorie-restricted diets had a greater impact on insulin sensitivity than exercise alone [20]. However, many diabetic patients or NAFLD patients failed to achieve clinical goals in clinical practice through calorie-restricted diets and/or PA due to poor adherence.

Metformin is the most common anti-hyperglycemic agent. Many studies have reported benefits of metformin in obesity, cardiovascular disease (CVD), and liver disease. Metformin treatment prevents obesity induced by a high-fat diet [21]. In humans, metformin decreased the incidence of CVD [22]. It has been shown that metformin reduced hepatocellular carcinoma incidence and improved survival [23]. Metformin caused a reduction of liver triglyceride content in ob/ob mice [24]. The benefits of metformin in treating NAFLD were also observed in human research [25]. But there is still controversy [26]. A randomized-controlled study of metformin monotherapy was rarely conducted in the NAFLD population with diabetes. Evidence related to the effectiveness of metformin in NAFLD patients with newly diagnosed T2DM is still insufficient.

Studies indicated that lifestyle modification (diet and physical activity interventions) regulated AMP-activated protein kinase (AMPK), a key molecular player linked with the function of energy metabolism [27]. It has been reported that metformin could regulate hepatic glucose output and lipid metabolism through the AMPK-dependent pathway [28]. In addition, recent evidence has shown that metformin may exert its antidiabetic effects by affecting the composition and function of the gut microbiota [29]. Diet and physical activity interventions have shown a potential role in modulating gut microbial balance [30]. As mentioned above, lifestyle modification and metformin may share the same biological target in the management of metabolic diseases. It has not been reported whether the superposition of these two interventions can be synergistic. Thus, we set up a group providing both lifestyle modification and metformin in our study.

There are several limitations in our study. First, the adherence for longtime lifestyle intervention may not be high. To minimize bias, we make regular return visits and strengthen education. Second, the self-reported dietary intake is subject to bias and limitations. Third, some subjects may be intolerant to metformin.

In summary, this study will assess the synergistic effect of intensive lifestyle combined with metformin on glycemic control, hepatic steatosis, and fibrosis.

## Trial status

This trial was registered on 31 May 2023. This study is under participant recruitment. The first patient was included on 6 June 2023. And the recruitment will be completed in December 2024.

## Supplementary Information

Supplementary Material 1. Table S1. Outcome definition.

## Data Availability

The initial trial data will be available to principal researchers or on demand from the corresponding author.

## Abbreviations

NAFLD: Nonalcoholic fatty liver disease
T2DM: Type 2 diabetes
HbA1c: Hemoglobin A1c
HOMA-IR: Homoeostasis model-insulin resistance index
BMI: Body mass index
WC: Waist circumference
LSM: Liver stiffness measurements
CAP: Controlled attenuation parameter
NASH: Nonalcoholic steatohepatitis
HCC: Hepatocellular carcinoma
PA: Physical activity
