# Supplementary material for: Randomized controlled trial of effects of metformin in NAFLD patients with newly diagnosed type 2 diabetes treated with an intensive lifestyle: a study protocol
Source: Trials. 2025 Oct 31;26:462. doi: 10.1186/s13063-025-09191-0 (PMC12577026; doi:10.1186/s13063-025-09191-0)
Supplement: Supplementary file 1 — Supplementary Material 1. Table S1. Outcome definition. [file 13063_2025_9191_MOESM1_ESM.docx]

Supplementary Tables S1

| **primary outcome** | Speccific measurement | metric | Method of aggregation | Time point |
| --- | --- | --- | --- | --- |
| Severity of Non-Alcoholic Fatty Liver Disease (NAFLD) | Hetatic steatosis  and fibrosis | Categorical scale (e.g., 0–3 for steatosis, 0–4 for fibrosis) | Proportion of participants in each severity category or median score with interquartile range (IQR) | baseline and 12 weeks post-intervention |
| **Secondary outcomes** |  |  |  |  |
| Insulin Resistance (HOMA-IR) | HOMA-IR index (calculated from fasting glucose and insulin) | Unitless score (e.g., HOMA-IR = [fasting glucose (mmol/L) × fasting insulin (mU/mL)] / 22.5) | Mean ± standard deviation (SD) or median with IQR | baseline and 12 weeks post-intervention |
| Glycemic Control (HbA1c) | HbA1c level (glycated hemoglobin) | Percentage (%) or mmol/mol | Mean ± SD or median with IQR |  |
| fasting blood glucose | Fasting plasma glucose concentration | mmol/L | Mean ± SD or median with IQR | baseline and 12 weeks post-intervention |
| 2-hour postprandial blood glucose | Plasma glucose concentration 2 hours after a standardized meal | mmol/L | Mean ± SD or median with IQR | baseline and 12 weeks post-intervention |
| Anthropometric Measures (BMI) | Body Mass Index (weight/height²) | kg/m² | Mean ± SD or median with IQR | baseline and 12 weeks post-intervention |
| Anthropometric Measures (Waist Circumference) | Mid-axillary waist circumference | cm | Mean ± SD or median with IQR | baseline and 12 weeks post-intervention |
| Lipid Profile (LDL-Cholesterol) | Low-density lipoprotein cholesterol | mmol/L | Mean ± SD or median with IQR | baseline and 12 weeks post-intervention |
| Lipid Profile (HDL-Cholesterol) | High-density lipoprotein cholesterol | mmol/L | Mean ± SD or median with IQR | baseline and 12 weeks post-intervention |
| Lipid Profile (Triglycerides) | Triglyceride concentration | mmol/L | Mean ± SD or median with IQR | baseline and 12 weeks post-intervention |
| Lipid Profile (Total Cholesterol) | Total cholesterol concentration | mmol/L | Mean ± SD or median with IQR | baseline and 12 weeks post-intervention |
| Cardiovascular Risk (Blood Pressure) | systolic and diastolic blood pressure | mmHg | Mean ± SD or median with IQR | baseline and 12 weeks post-intervention |
